# Efficacy and cost-effectiveness of a novel dual gasping forceps-assisted over-the-scope clip inverted closure after gastric endoscopic full-thickness resection

**DOI:** 10.1055/a-2108-1037

**Published:** 2023-07-11

**Authors:** Noriko Nishiyama, Shintaro Fujihara, Naoya Tada, Kaho Nakatani, Asahiro Morishita, Takayoshi Kishino, Hideki Kobara

**Affiliations:** 1 Department of Gastroenterology and Neurology, Faculty of Medicine, Kagawa University, Kagawa, Japan; 2 Department of Gastroenterological Surgery, Faculty of Medicine, Kagawa University, Kagawa, Japan

Achieving reliable full-thickness defect closure after gastric endoscopic full-thickness resection (EFTR) for gastrointestinal stromal tumors is challenging 1 2 . Although mucosal closure appears clinically acceptable 2 3 4 , it is crucial to develop a robust and technically easy closure method that enables serosa-serosa inverted closure, as with surgical sutures. Here, we describe a novel dual slim grasping forceps-assisted over-the-scope (OTS) clip closure under dual-channel endoscopy in gastric EFTR. With this technique, the difficult maneuvers and high cost associated with Twin Grasper forceps (Ovesco Endoscopy GmbH, Tübingen, Germany) can be overcome.

A 65-year-old man presented with a gastrointestinal stromal tumor in the upper stomach. After standard EFTR, a full-thickness defect measuring 15 mm in diameter remained ( Fig. 1 a ). After obtaining written informed consent, the defect was closed according to the following description ( Fig. 2 , Video 1 ). The equipment comprised two grasping forceps (TechGrasper; Micro-Tech, Nanjing, China) (cost US$24) instead of the Twin Grasper forceps (cost US$ 694). TechGrasper forceps have two advantages: the slim shaft (1.8 mm outer diameter) is less likely to interfere with suction power before firing the OTS clip, and the deep spike provides strength when grasping the seromuscular layer. The TechGrasper forceps were inserted into the gastroscope’s dual channels (GIF-2TQ260M; Olympus, Tokyo, Japan) mounted with an OTS clip (gc type, 10 mm; Ovesco Endoscopy). One forceps was used to grasp the seromuscular layer on one side of the defect, and the other was opened to grasp the contralateral seromuscular layer ( Fig. 1 b ). Both grasping forceps were pulled into the OTS clip cap under sufficient suction, and an OTS clip was deployed ( Fig. 1 c ). The seromuscular layer was inverted in tight apposition. The closure time was 30 minutes. Laparoscopic observation revealed no air leakage and the inverted full-thickness closure was confirmed ( Fig. 3 ).

**Fig. 1:**
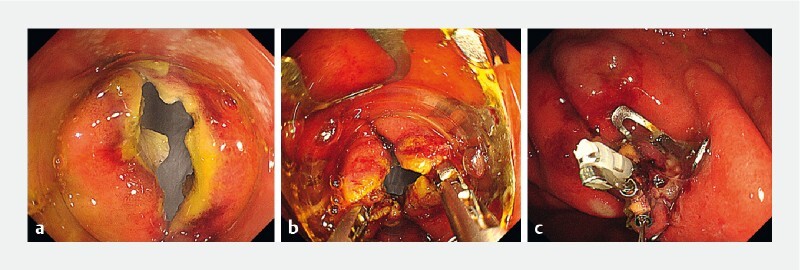
Endoscopic images. **a** After standard full-thickness resection, a defect measuring 15 mm in diameter remained. **b** Two grasping forceps were inserted into the gastroscope’s dual channels mounted with an over-the-scope (OTS) clip, and the seromuscular layers on both sides of the defect were grasped. **c** The two grasping forceps were pulled into the OTS clip cap under sufficient suction, and the OTS clip was deployed.

**Fig. 2:**
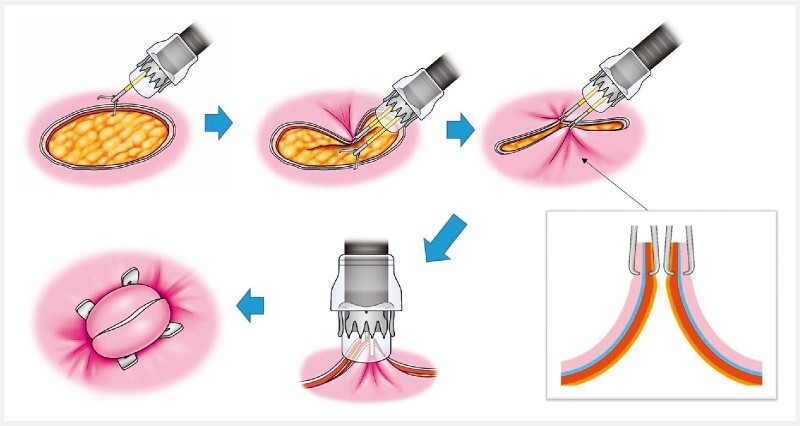
Schema showing over-the-scope clip closure using dual grasping forceps (TechGrasper; Micro-Tech, Nanjing, China). Source: Davinchi Medical Illustration Office.

**Video 1** Over-the-scope clip closure using the dual TechGrasper (Micro-Tech, Nanjing, China) after endoscopic full-thickness resection.


**Fig. 3:**
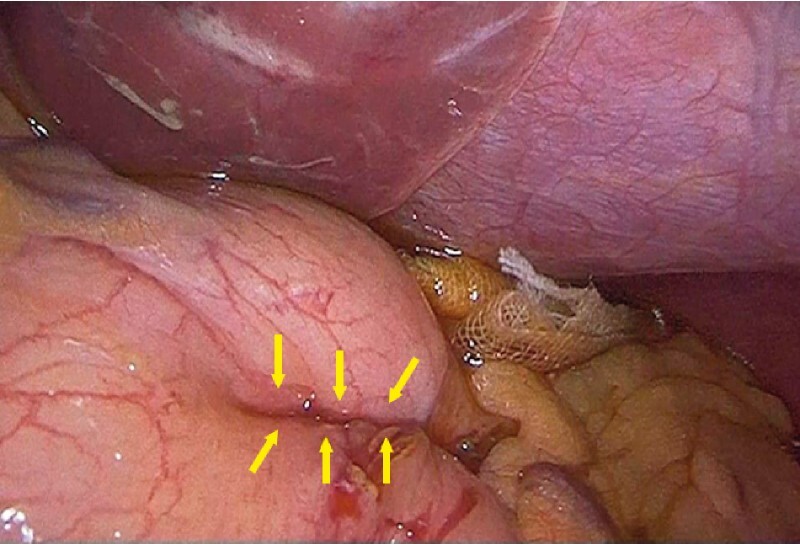
Laparoscopic view showing that the seromuscular layer was inverted in tight apposition. Yellow arrows show the closure line.

Dual TechGrasper-assisted OTS clip closure is cost-effective and a useful option for facilitating inverted full-thickness closure.

Endoscopy_UCTN_Code_TTT_1AO_2AG

## Notes

Endoscopy E-Videos

https://eref.thieme.de/e-videos
*E-Videos* is an open access online section of the journal *Endoscopy* , reporting on interesting cases and new techniques in gastroenterological endoscopy. All papers include a high-quality video and are published with a Creative Commons CC-BY license. Endoscopy E-Videos qualify for HINARI discounts and waivers and eligibility is automatically checked during the submission process. We grant 100% waivers to articles whose corresponding authors are based in Group A countries and 50% waivers to those who are based in Group B countries as classified by Research4Life (see: https://www.research4life.org/access/eligibility/ ). This section has its own submission website at https://mc.manuscriptcentral.com/e-videos
