# Correction: Efficacy and cost-effectiveness of a novel dual grasping forceps-assisted over-the-scope clip inverted closure after gastric endoscopic full-thickness resection

**DOI:** 10.1055/a-2155-7593

**Published:** 2023-08-22

**Authors:** Noriko Nishiyama, Shintaro Fujihara, Naoya Tada, Kaho Nakatani, Asahiro Morishita, Takayoshi Kishino, Hideki Kobara

**Affiliations:** 1 Department of Gastroenterology and Neurology, Faculty of Medicine, Kagawa University, Kagawa, Japan; 2 Department of Gastroenterological Surgery, Faculty of Medicine, Kagawa University, Kagawa, Japan

**Correction**

**Correction: Efficacy and cost-effectiveness of a novel dual grasping forceps-assisted over-the-scope clip inverted closure after gastric endoscopic full-thickness resection** Nishiyama N, Fujihara S, Tada N et al. Efficacy and cost-effectiveness of a novel dual grasping forceps-assisted over-the-scope clip inverted closure after gastric endoscopic full-thickness resection. Endoscopy 2023; 55: E870–E871, doi:10.1055/a-2108-1037 In the above-mentioned article, the title has been corrected. Correct is: Efficacy and cost-effectiveness of a novel dual grasping forceps-assisted over-the-scope clip inverted closure after gastric endoscopic full-thickness resection. This was corrected in the online version on August 22, 2023.

